# Taking Risks With Cybersecurity: Using Knowledge and Personal Characteristics to Predict Self-Reported Cybersecurity Behaviors

**DOI:** 10.3389/fpsyg.2020.546546

**Published:** 2020-11-04

**Authors:** Shelia M. Kennison, Eric Chan-Tin

**Affiliations:** ^1^Department of Psychology, Oklahoma State University, Stillwater, OK, United States; ^2^Department of Computer Science, Loyola University Chicago, Chicago, IL, United States

**Keywords:** risk-taking, cybersecurity, passwords, personality, DOSPERT

## Abstract

Individuals’ use of insecure cybersecurity behaviors, including the use of weak passwords, is a leading contributor to cybersecurity breaches. While training individuals on best practices in cybersecurity continues to be implemented, prior research has found that training people in the use of secure passwords has not proven to be effective. Developing profiles of individual who are likely to become victims of password hacking, phishing scams, and other types of breaches would be useful, as they could be used to identify individuals with the highest likelihood of engaging in insecure cybersecurity behaviors. The present research tested the hypothesis that in addition to self-reported cybersecurity knowledge, personal characteristics, such as personality traits and general risk-taking behavior not related to technology use, can predict individual differences in cybersecurity behaviors, as measured by self-report. Our hypothesis was confirmed in a large study involving 325 undergraduates. Participants provided information about their self-reported risky cybersecurity behaviors (e.g., using non-secure Wi-Fi, not logging out of accounts on shared computers, etc.), self-reported knowledge about strong/weak passwords, Big Five personality traits (i.e., extraversion, conscientiousness, agreeableness, openness, and mood instability), sensation-seeking personality traits, and general risk-taking unrelated to using technology. The results of a hierarchical regression indicated that 34% of risky cybersecurity behavior was significantly predicted by the combination of self-reported knowledge about strong/weak passwords, personality traits, and risk-taking in daily life. The results suggest that victim profiles should take into account individual differences in personality and general risk-taking in domains unrelated to cybersecurity in addition to cybersecurity knowledge.

## Introduction

The average American has little awareness of cybersecurity issues, despite the fact that the majority have been affected by some type of security breach (Pew Research Center, 2017). Research has documented that using weak passwords and re-using passwords for multiple accounts is common (Gaw and Felten, 2006; Florencio and Herley, 2007; Grawemeyer and Johnson, 2011). Recent research has explored strategies for reducing computer users’ vulnerabilities by educating them about the dangers of risky cybersecurity behaviors, such as choosing weak passwords (Farcasin and Chan-Tin, 2015) and re-using passwords (Stobert and Biddle, 2014). Research has shown that educating people on security best practices through trainings may not be effective (Riley, 2006; Lorenz et al., 2013). Studies have shown that those with knowledge about password security will, nonetheless, use weak passwords and/or re-use passwords in their daily lives (Riley, 2006; Notoatmodjo and Thomborson, 2009). Nevertheless, continued efforts to develop and to test the effectiveness of training curriculum are warranted (Bryant and Campbell, 2006; Taylor-Jackson et al., 2020). Few studies have investigated whether personality traits predict knowledge and cybersecurity behaviors (e.g., Whitty et al., 2015). The focus of the present research was to determine whether risky cybersecurity behavior could be predicted from a combination of password security knowledge and personal characteristics, such as personality traits and general risk-taking in daily life.

Case studies of cybersecurity breaches have shown that humans, rather than technology, are the *weakest link*, responsible for risky cybersecurity behaviors that provide access points for cybersecurity attacks (Mitnick, 2003; Pew Research Center, 2017; cf. Adams and Sasse, 1999). Numerous security breaches have involved the use of weak passwords. Some examples include the credit report company Equifax (Wang and Johnson, 2018), the retailer Target (Plachkinova and Maurer, 2019), and an American university (Ayyagari and Tyks, 2012). An increasing number of platforms are implementing requirements for users to use stronger passwords (i.e., with a combination of numbers, lowercase and uppercase letters, and other symbols or a passphrase); however, security vulnerability remains when users use the same password for multiple accounts (Thomas et al., 2017).

Numerous studies have explored the effectiveness of cybersecurity training to increase users’ knowledge about best cybersecurity practices and to decrease the use of risky cybersecurity behaviors in daily life (Ferguson, 2005; McCrohan et al., 2010; Peker et al., 2016; see for review, Proctor, 2016). A prevalent view is that institutions should not rely solely on cybersecurity training, because in the past, it has not been shown to be effective (Ferguson, 2005; Bada et al., 2019). There is also the recognition that regardless of how much training an institution carries out, a security breach can occur from a small number of individuals’ risky cybersecurity behaviors. In a large sample of student participants, Riley (2006) showed that individuals may use weak passwords despite reporting that they knew that such passwords were not the most secure. Nevertheless, several recent studies have demonstrated some positive benefits of training (McCrohan et al., 2010; Peker et al., 2016). The trainings that have shown benefits have focused on providing individuals with knowledge about cybersecurity threats and best cybersecurity practices. Adams and Sasse (1999) suggested that users’ lack of knowledge about cybersecurity and their perceptions of insecure practices as low-risk may be due to inadequate communication to users from the relevant institutional entities.

The present research examined the possibility that it may be possible to predict individual differences in risky cybersecurity behaviors using personal characteristics, such as knowledge about password security, personality traits, and personality-related behaviors. Prior research has found that men report higher levels of knowledge about cybersecurity than women (Cain et al., 2018) and also higher levels of risky cybersecurity behavior (Anwar et al., 2017). Numerous studies have examined the relationships among Big Five personality traits (i.e., extraversion, agreeableness, openness, conscientiousness, and mood instability) and cybersecurity behavior (McBride et al., 2012; Tamrakar et al., 2016; McCormac et al., 2017; Russell et al., 2017; Alohali et al., 2018; Shappie et al., 2019). These studies have looked at the relationship between personality traits and cybersecurity behavior. Big five personality traits have been described as universal (Yamagata et al., 2006; cf. Gurven et al., 2013) and stable across the lifespan (Conley, 1985). Tamrakar et al. (2016) created a simulation tool to measure the relationship between personality traits and cyber behaviors. Russell et al. (2017) studied how people engaged in secure cybersecurity behaviors are more positive. They also found that secure cybersecurity behaviors are linked to emptiness and meaningless while greater use of insecure cybersecurity behaviors are related to lower conscientiousness and higher levels of aggressive behavior.

Some studies are slightly contradicting. Shappie et al. (2019) showed that conscientiousness, agreeableness, and openness were significantly associated with cybersecurity behaviors. In contrast, Alohali et al. (2018) showed that conscientiousness is negatively correlated with cybersecurity behaviors. The Human Aspects of Information Security Questionnaire (HAIS-Q) was utilized in McCormac et al. (2017); they have shown that conscientiousness, agreeableness, emotional stability, and risk-taking propensity significantly explained the variance in individuals’ score, while age and gender did not. While most papers recruited participants from schools (McBride et al., 2012), recruited IT practitioners and looked at how likely these practitioners are to violate cybersecurity protocols based on their Big Five personality traits. In a recent study by Maraj et al. (2019), there was no relationship found between password strength and personality traits.

In addition to examining the relationships among the Big Five traits and risky cybersecurity behaviors, we also examined sensation-seeking personality traits and the extent to which individuals take risks in daily life that were unrelated to the use of technology. Sensation-seeking personality traits were first identified by Zuckerman et al.(1964,1978; Horvath and Zuckerman, 1993; Zuckerman, 1994) and defined as the propensity to seek out new experiences with a preference toward intense experiences. Numerous studies have shown that individuals higher in sensation-seeking traits take more risks in daily life, including participating in sports (Zuckerman, 1983a), smoking, drinking and using other drugs (Zuckerman, 1983b, 1987; Zuckerman et al., 1990; Popham et al., 2011; Kennison and Messer, 2017, 2019), engaging in risky sexual behaviors (Zuckerman et al., 1976), and risky behaviors occurring during gambling (Anderson and Brown, 1984). Numerous studies suggest that sensation-seeking traits stem from individual differences in biology (see for review Roberti, 2004).

We reasoned that individuals with higher levels of sensation-seeking personality traits would engage in higher levels of risky cybersecurity behaviors and those who engage in higher levels of general risk-taking in daily life would engage in higher levels of risky cybersecurity behavior. To measure general risk-taking in daily life, we used the Domain-Specific Risk Taking (DOSPERT) scale (Blais and Weber, 2001, 2006; Weber et al., 2002; Figner and Weber, 2011), which assesses risk-taking in five domains: (a) health/safety, (b) recreational, (c) social, (d) financial, and (e) ethical. Multiple studies in which the scale was used have shown that there are significant correlations for risk-taking for the five domains, suggesting that high risk-taking in one domain predicts high risk-taking in the other domains (Kennison et al., 2016; Shou and Olney, 2020). Shou and Olney (2020) carried out a large meta-analysis using 104 samples with more than 30,000 observations and found that the five domains were intercorrelated. The health/safety domain was strongly correlated with recreational and ethical domains. The social domain was more weakly correlated with the other domains. Prior research has also observed differences in the perception of risk for men and women, with women perceiving more risk generally than men and being more risk-adverse (Gustafsod, 1998; Weber et al., 2002). Men also report engaging in risk-taking in daily life more than women (Kennison et al., 2016; see Panno et al., 2018 for review). Men also report higher levels of sensation-seeking traits than women (Kennison et al., 2016).

In this paper, we report a study that was carried out online in which we investigated how well self-reported opinion about knowledge about secure passwords, personality traits, and general risk-taking in daily life predict self-reported risky cybersecurity behaviors. Increasingly, researchers are carrying out research via the Internet (Buchanan and Smith, 1999; Gosling et al., 2004; Weigold et al., 2013; Dodou and de Winter, 2014), which leads to lower costs as staff are not needed for data entry after study completion. Internet research has been positively impacted by the increasing availability of Internet access and inexpensive survey building tools. Confidence in online research has grown due to studies that have compared data collected via the Internet and in face-to-face settings in which questionnaires were completed using pencil/paper methods and have concluded that the two data collection methods yield similar results (Gosling et al., 2004; Weigold et al., 2013). Differences in response rates, amount of missing data, and factor structure of some variables have been observed (see Weigold et al., 2013 for review). Some have suggested that participants in studies carried out via the Internet may differ in their tendency to provide socially desirable responses than participants in studies carried out in face-to-face settings (see Dodou and de Winter, 2014 for review). Dodou and de Winter (2014) carried out a meta-analysis of 51 prior research studies in which social desirability responding was compared for online and face-to-face studies. In the meta-analysis, they found that social desirability responding was similar for the two methodologies. Others have suggested that in some cases, participants may be willing to be more truthful in responding in online surveys versus studies conducted in face-to-face settings (Bailey et al., 2000).

In our study, we tested the following hypotheses: (a) higher levels of self-reported password security knowledge would be related to engaging in lower levels of risky cybersecurity behaviors (see Ferguson, 2005; McCrohan et al., 2010; Peker et al., 2016), (b) higher levels of conscientiousness will be related to lower levels of risky cybersecurity behaviors (see McCormac et al., 2017; Russell et al., 2017; Alohali et al., 2018; Shappie et al., 2019), (c) higher levels of mood instability will be related to higher levels of self-reported risky cybersecurity behavior (see McCormac et al., 2017), (d) higher levels of sensation-seeking will be related to higher levels of self-reported risky cybersecurity behaviors (cf. Whitty et al., 2015), (e) higher levels of general risk-taking behaviors will be related to higher levels of risky cybersecurity behaviors, and (f) men would engage in higher levels of risk-taking (i.e., general risk-taking and risky cybersecurity behavior) than women. We did not expect to observe significant relationships between personality traits and knowledge, as prior research has not provided evidence for these relationships and also because gaining knowledge able password security would not be expected to depend on personality traits. Knowledge is gained through communications schools or workplaces, which are experienced by people regardless of their personality traits.

## Materials and Methods

### Participants

There were 325 participants (207 women, 117 men, and 1 *other*) who were taking classes in psychology or speech communications a large public university in the Midwestern region of the United States. All participants received credit that could be used for course requirements or extra credit. Participants were on average 19.46 years old (*SD* = 2.34).

### Procedure and Materials

This study was carried out in accordance with the recommendations of Oklahoma State University’s Institutional Review Board (IRB), which approved the protocol. After obtaining IRB approval for the study, we recruited volunteers from a research participant SONA pool in a psychology department. In the SONA recruitment description for the study, participants were told that the purpose of the study was “The purpose of this research is to investigate the relationship between password security beliefs and behaviors with personality and demographic variables.” All participants gave informed consent in accordance with the Declaration of Helsinki. As recommended for all surveys conducted via the Internet (Kraut et al., 2004), the first page of our survey provided participants with information about the study and an opportunity to volunteer for the study. The research was conducted with a waiver of documentation of consent, which is common with surveys conducted over the Internet. Participants completed an online survey created using a Professional license of Surveymonkey.com. On the first page of the survey, participants viewed information about the study and instructions on how to volunteer or to decline to volunteer. All participants completed a survey in the same order. The following order was used: Big Five personality traits, sensation-seeking personality, general risk-taking in daily life, cybersecurity behavior, knowledge, and demographics. On average, participants took 37 min to complete the survey.

We assessed participants’ use of risky cybersecurity practices using six items created for the present research. We considered some of the most common risk cybersecurity behaviors that would be relevant to young adults in a college setting relying on direction from prior research (Peker et al., 2016; Ramlo and Nicholas, 2020). We generated six items focused on a situation that would likely be familiar to most students on our campus. Each item addressed one cybersecurity behavior. The prior literature identified more than six problem behaviors. We chose the six problem behaviors that we believed would likely be familiar to most student on our campus and carried out a focus group of undergraduates who did not participate in the study. We confirmed from the group that the behavior would likely be familiar to most of their peers. In the survey, each item was paired with a 7-point scale (1 = *not at all likely* and 7 = *extremely likely*). The scale numbers in-between were not labeled. Each item described a practice that should be avoided. The items were: (a) *using weak passwords (e.g., pass1234)*, (b) *failing to log out of a shared computer, such as in a campus computer lab*, (c) *clicking on an unfamiliar URL link that you receive in an email*, (d) *using public unsecured Wi-Fi*, (e) *using the same password for multiple devices/applications*, and (f) *telling your password to someone at your workplace*. Items were presented in random order for each participant. We computed the mean rating for the six items with higher means reflecting higher levels of secure self-reported behavior. We observed good internal consistency for the four items (Cronbach α = 0.77, see Taber, 2018 for discussion of importance of internal consistency in psychometric measures). Nunnally (1978) suggests values above 0.70 reflect good internal consistency. Below 0.70 is viewed as questionable (George and Mallery, 2003).

We assessed participants’ rating of their opinion of their own knowledge of password security using four items created for this study. Each item was paired with a 7-point scale (1 = *Strongly Disagree* and 7 = *Strongly Agree*). The scale number in-between were also labeled (i.e., 2 = *Moderately Disagree*, 3 = *Somewhat Disagree*, 4 = *Neither Disagree nor Agree*, 5 = *Somewhat Agree*, and 6 = *Moderately Agree*). The questions were: (a) *My knowledge of password security is high*, (b) *Password security practices are not something that I have learned very much about*, (c) *I know a lot about password security practices*, and (d) *My level of knowledge about real world cases where sensitive data have been stolen by hackers is fairly high*. Items were presented in random order. After reverse scoring the second item in the above sequence, a mean score for the four items was calculated. We observed good internal consistency for the four items (Cronbach α = 0.74; see Taber, 2018). The items contain some overlap. We examined correlations with subsets of the items and found similar results as when all items were used. We are reporting the results for all the items for this reason.

Sensation-seeking personality traits were assessed using Zuckerman et al. (1978) 40-item SSS-V Scale. The SSS-V is composed of four factors: (a) thrill and adventure seeking (TAS, i.e., affinity for participating in activities characterized as dangerous), (b) experience seeking (ES, i.e., interest in seeking out new experiences including unusual lifestyle practices), (c) disinhibition (DIS, i.e., affinity for out-of-control experiences, such as those that occur experiences with drugs, parties, or sexual interactions), and (d) boredom susceptibility (BS, i.e., dislike of feeling bored, including being around people who are boring). For each of the 40 items, participants viewed two statements and were asked to choose the one that best described them [e.g., (a) *I like “wild” uninhibited parties*. vs. (b) *I prefer quiet parties with good conversation*]. Prior research has demonstrated the validity of the scale (Zuckerman and Link, 1968). Prior research has shown that these factors have good internal consistency; the Cronbach alphas for the four factors: TAS (α = 0.78), DIS (α = 0.76), ES (α = 0.72), and BS (α = 0.74) (Kennison et al., 2016). In the present research, we also observed good internal consistency for the four factors with Cronbach values ranging from α = 0.72 to α = 0.78 (see Nunnally, 1978; George and Mallery, 2003; Taber, 2018).

We assessed risk-taking in daily life using Blais and Weber’s (2006) 30-item DOSPERT. The scale is composed of five types of risk-taking: health (i.e., risk-taking in the form of careless as well as abuse of drugs), recreational (i.e., risk-taking when doing sports and other recreational activities), social (taking risks with social interactions, such as risky behaviors with superiors), financial (i.e., risk-taking with money), and ethical (i.e., engaging in criminal behavior as well as lying and cheating). The 30-items are specific behaviors, and participants rate on a 7-point rating scale ranging from 1 (*Extremely Unlikely*) to 7 (*Extremely Likely*) how likely they are to engage in the behaviors. The scale number in-between were also labeled (i.e., 2 = *moderately unlikely*, 3 = *somewhat unlikely*, 4 = *neither unlikely nor likely*, 5 = *somewhat likely*, and 6 = *moderately likely*). Prior research has demonstrated the validity of the scale (Frey et al., 2017) and has shown that the five domains have good internal consistency: (a) health (α = 0.76), (b) recreational (α = 0.84), (c) social (α = 0.71), (d) financial (α = 0.84), and ethical (α = 0.83) (Kennison et al., 2016). In the present research, we also observed good internal consistency for the five domains with Cronbach alphas ranging from α = 0.71 to α = 0.84 (see Nunnally, 1978; George and Mallery, 2003; Taber, 2018).

We asked participants about their Big 5 personality traits (i.e., extraversion, agreeableness, conscientiousness, openness, and mood instability) using Saucier’s (1994) Mini-Marker measure, which contains 40 adjectives (i.e., 8 for each trait). Participants are asked how accurate each adjective is in describing them using a 9-point scale (1 = *extremely inaccurate* and 9 = *extremely accurate*). The scale number in-between were also labeled (i.e., 2 = *very inaccurate*, 3 = *moderately inaccurate*, 4 = *slightly inaccurate*, 5 = *neither accurate nor inaccurate*, 6 = *slightly accurate*, 7 = *moderately accurate*, and 8 = *very accurate*). After reverse scoring when appropriate, we calculated the average rating for the eight adjectives for each trait. The validity of the measure has been demonstrated in prior research (Dwight et al., 1998). The measure is associated with high internal consistency (Cronbach alphas between from 0.76 to 0.86, Mooradian and Nezlek, 1996). We also observed high internal consistency in the present study (Cronbach alphas between α = 0.69 and α = 0.82; see Nunnally, 1978; George and Mallery, 2003; Taber, 2018).

One question was included as an attention check, assessing participants’ attention to the survey with a 5-point response scale (1 = *strongly disagree*, 2 = *slightly disagree*, 3 = *neither disagree nor agree*, 4 = *slightly agree*, and 5 = *strongly agree*). Each option was listed on a separate line in multiple choice format. The question text was as follows: *Sometimes researchers include a question to determine if the participant is paying adequate attention while completing the survey. In order to show us that you are paying attention please select the third option as the response to this question*.

## Results

The dataset including participants’ responses was screened to detect any participants who incorrectly responded to the attention check question. Thirty-three participants were removed from the dataset. The resulting dataset contained data from 292 participants (186 women, 105 men, and 1 who selected *other* for gender). Table 1 displays means, standard deviations, and Pearson’s *r* product-moment correlations for the variables that we measured in the study. Prior to conducting the correlations, we examined ranges for all variables and found no indication that there was restriction of range. The results indicated support or partial support for the four hypotheses: (a) higher levels of self-reported password security knowledge were related to lower levels of self-reported risky cybersecurity behaviors, (b) higher levels of sensation-seeking were related to higher levels of self-reported risky cybersecurity behaviors for women, but not men, (c) higher levels of mood instability were related to higher levels of self-reported risky cybersecurity behaviors, (d) higher levels of general risk-taking behaviors were related to higher levels of self-reported risky cybersecurity behaviors, (e) higher levels of conscientiousness were related to lower levels of risky cybersecurity behaviors for women, but not men, and (f) men reported engaging in higher levels of general risk-taking than women, *t*(286) = −5.54, *p* < 0.001, and η^2^ = 0.41l, but there was no significant difference in self-reported risky cybersecurity behavior for men and women. Contrary to expectations, we found that for both men and women, higher levels of mood instability predicted higher levels of self-reported risky cyber security behavior. For the remaining three of the five personality traits, none were related to self-reported cybersecurity knowledge or self-reported risky cybersecurity behaviors. In addition, we found that compared to women, men reported having higher levels of knowledge of secure passwords, *t*(287) = −2.02, *p* = 0.04, and η^2^ = 0.09; lower levels of conscientiousness, *t*(288) = 3.20, *p* = 0.002, and η^2^ = 0.22; lower levels of extraversion, *t*(288) = 2.02, *p* = 0.04, and η^2^ = 0.22; lower levels of agreeableness, *t*(288) = 3.33, *p* = 0.001, and η^2^ = 0.20; and higher levels of sensation-seeking personality, *t*(289) = −5.08, *p* < 0.001, and η^2^ = 0.17.

**TABLE 1:** Summary of descriptive statistics and correlations for men (lower half of matrix) and women (upper half of matrix).

Variable	1	2	3	4	5	6	7	8	9	Mean	SE
1. Risky cybersecurity behavior	–	−0.26***	−0.01	−0.27***	−0.14	−0.10	0.16*	0.49***	0.15*	2.92	0.09
2. Secure password knowledge	−0.25*	–	0.04	0.08	−0.06	0.10	0.08	−0.05	0.01	3.79	0.09
3. Extraversion	–0.01	–0.03	–	0.03	0.21**	0.10	0.02	0.32***	0.25***	5.88	0.10
4. Conscientiousness	–0.05	–0.01	0.36***	–	0.41***	0.24***	−0.27***	−0.26***	−0.20**	6.44	0.08
5. Agreeableness	–0.03	0.13	0.19	0.24*	–	0.32***	−0.39***	−0.18*	−0.13	7.04	0.09
6. Openness	0.07	0.04	0.22*	0.27**	0.34***	–	-0.01	0.07	0.23**	6.19	0.08
7. Mood instability	0.21*	–0.14	0.07	0.06	−0.22*	0.03	–	0.05	0.04	4.51	0.09
8. General risk-taking (DOSPERT)	0.47***	0.12	0.19	−0.10	−0.12	0.10	+ 0.03	–	0.50***	2.75	0.06
9. Sensation seeking personality	–0.10	0.12	0.19	0.09	−0.13	0.00	0.14	0.20*	–	16.18	0.45
Mean	2.86	4.10	5.52	5.99	6.58	6.40	4.31	3.32	19.72		
SE	0.10	0.12	0.14	0.11	0.11	0.11	0.10	0.08	0.49		

Lower half of the matrix provides results for men and upper half provides results for women. *p < 0.05, **p < 0.01, ***p < 0.001. Bolded correlations are statistically significant.

To investigate further how self-reported knowledge about strong/weak passwords, personality traits, general risk-taking, and predict risky cybersecurity behaviors, we carried out a hierarchical multiple regression using risky cybersecurity behaviors as the dependent variable and four blocks of variables. Variables were examined to confirm that assumptions of normality, linearity, and homoscedasticity were met (Hair et al., 1998). We ordered the variables with a developmental trajectory of the individual in mind with personal characteristics entered in early blocks and self-reported knowledge and self-reported cybersecurity-related behaviors, in later blocks. This enabled us to examine the results for knowledge and cybersecurity behavior while controlling for personal characteristics and to examine cybersecurity behavior, while controlling for knowledge (Keith, 2014). In block one, sex was entered to control for sex differences. Subsequent blocks involved personality variables before knowledge, as both Big Five personality traits and sensation-seeking personality are generally believed to develop early in life and have a basis in biology (Fulker et al., 1980; Jang et al., 1996, respectively) and knowledge about technology acquired later. In block two, Big Five personality traits were added. In block three, sensation-seeking personality traits and general risk-taking were added, and in block four, knowledge of password security was added. We found that variables did not involve excessive collinearity (as evidenced by the Tolerance and VIF values). Excessive collinearity would weaken the statistical power of the analysis (Coakes, 2005). Table 2 displays the summary of these results.

**TABLE 2:** Summary of hierarchical regression analysis for variables predicting lax cybersecurity behaviors.

Variable	β	*T*	sr^2^	*R*	*R*^2^	Δ*R*^2^
Block 1				0.004	0.00	0.00
Sex	−0.004	−0.07	0.00			
Block 2				0.25	0.04	0.06**
Sex	−0.02	−0.36	0.00			
Conscientiousness	−0.19	−2.94**	0.03			
Extraversion	0.01	0.10	0.00			
Agreeableness	0.04	0.56	0.01			
Openness	−0.02	−0.27	0.00			
Mood instability	0.16	2.58*	0.02			
Block 3				0.54	0.28	0.23***
Sex	−0.15	−2.56*	0.02			
Conscientiousness	−0.08	−1.30	0.00			
Extraversion	−0.14	−2.46*	0.02			
Agreeableness	0.13	2.02	0.01			
Openness	−0.08	−1.38	0.01			
Mood instability	0.17	3.16**	0.03			
Sensation-seeking traits	−0.13	−2.18*	0.01			
General risk-taking (DOSPERT)	0.59	9.54***	0.24			
Block 4				0.60	0.34	0.06***
Sex	−0.12	−2.19*	0.01			
Conscientiousness	−0.06	−1.10	0.00			
Extraversion	−0.14	−2.58*	0.02			
Agreeableness	0.12	2.03*	0.01			
Openness	−0.06	−1.12	0.00			
Mood instability	0.17	3.28***	0.03			
Sensation-seeking traits	−0.12	−2.12*	0.01			
General risk-taking (DOSPERT)	0.59	10.05***	0.24			
Password knowledge	−0.26	−5.23***	0.06			

*p < 0.05, **p < 0.01, ***p < 0.001.

In Block 1, participant sex did not significantly contribute to the variance in risky cybersecurity behaviors, *F*(1, 287) = 0.01 and *p* = 0.91. In Block 2, Big Five personality traits contributed significantly to variance in risky cybersecurity behaviors, accounting for 6% of the variance, *F*(6, 287) = 3.11 and *p* = 0.006 and the change in *R*^2^ was significant, *F*(5, 287) = 3.73 and *p* = 0.006. Two of the five traits were significant predictors: (a) conscientiousness (β = −0.19 and *p* = 0.03) and (b) mood instability (β = 0.16 and *p* = 0.02). In Block 3, sensation-seeking personality traits and general risk-taking accounted for an additional 28% of variance in risky cybersecurity behaviors, *F*(8, 287) = 13.70 and *p* < 0.001, and the change in *R*^2^ was significant, *F*(2, 287) = 47.30 and *p* < 0.001. Both variables were significant predictors: (a) sensation-seeking personality traits (β = −0.14 and *p* = 0.02) and (b) general risk-taking (β = 0.59 and *p* < 0.001). In Block 4, knowledge about secure passwords accounted for an additional 6% of variance in risky cybersecurity behaviors, *F*(9, 287) = 17.48 and *p* < 0.001, and the change in *R*^2^ was significant, *F*(1, 287) = 26.86 and *p* < 0.001. Knowledge about security passwords was a significant predictor (β = −0.25 and *p* < 0.001). The total amount of variance accounted was 34%.

## Discussion

The present research investigated how well self-reported risky cybersecurity behavior could be predicted by a combination of self-reported knowledge about secure passwords and personal characteristics, such as personality traits and general risk-taking in daily life. The majority of hypotheses tested were supported, including that (a) higher levels of self-reported password security knowledge was related to lower levels of self-reported risky cybersecurity behaviors, (b) higher levels of conscientiousness was related to lower levels of self-reported risky cybersecurity behaviors, (c) higher levels of mood instability was related to higher levels of self-reported risky cybersecurity behavior, (d) higher levels of sensation-seeking was related to higher levels of self-reported risky cybersecurity behaviors, (e) higher levels of general risk-taking behaviors was related to higher levels of risky cybersecurity behaviors, and (f) men reported engaging in more risk-taking in daily life than women, but the level of self-reported risky cybersecurity behavior did not differ for men and women. There were significant results that were not predicted. These include that for both men and women, higher levels of mood instability predicted higher levels of self-reported risky cyber security behavior; men reported having higher levels of password security knowledge than women.

The results showed that higher levels of self-reported password security knowledge was related to lower levels of self-reported risky cybersecurity behaviors, as has also been observed in prior research (Ferguson, 2005; McCrohan et al., 2010; Peker et al., 2016). Second, we found that women’s higher levels of sensation-seeking, but not men’s, were related to higher levels of self-reported risky cybersecurity behaviors for women. In prior research, sensation-seeking was not found to be related to cybersecurity behaviors (Whitty et al., 2015). Third, we found that higher levels of general risk-taking behaviors were related to higher levels of self-reported risky cybersecurity behaviors. Fourth, we found that conscientiousness predicted self-reported risky cybersecurity behaviors for women, but not men (cf. McCormac et al., 2017; Russell et al., 2017; Alohali et al., 2018; Shappie et al., 2019). The results also showed that higher levels of mood instability predicted higher levels of self-report risky cyber security behaviors, as has been observed in prior research (McCormac et al., 2017). We did not observe significant relationships between other three Big Five factors and risky cybersecurity behaviors. Our results showing that higher levels of sensation-seeking personality traits and general risk-taking in daily life predict greater use of risky cybersecurity behaviors are novel. These variables together contributed approximately 28% of the variance in cybersecurity behaviors, respectively. Overall, in our study in which 325 participants self-reported information about their password security knowledge, personality, risk-taking in daily life, and risky cybersecurity behavior, we found that personality variables and knowledge together predicted 34% of the variance in risky cybersecurity behaviors which exceeds the variance accounted for in prior research, which ranged between 3 and 5%.

The research has multiple strengths. It is the first to show that there is advantage to using personality traits in combination with other personal characteristics in predicting self-reported cybersecurity behavior. The statistical analysis provides estimates for the contributions of each. In addition, the present study is the first to show that general risk-taking in daily life predicts self-reported cybersecurity behavior. These results have implications for approaches in cybersecurity that involve training of individuals. These results suggest that creating profiles of potential victims of cybersecurity breaches should include personality variables, such as the Big Five and sensation-seeking, general risk-taking in daily life that is unrelated to using technology, in addition to knowledge about best practices in cybersecurity. The present results are the first to document that those who engage in higher levels of general risk-taking in daily life are also more likely to engage in risky cybersecurity behaviors. Our results suggest that accurate victim profiles could be useful in identifying individuals who are likely to be engaging in the highest levels of insecure cybersecurity behaviors. Institutions could use victim profiles to target such individuals with cybersecurity training that is in addition to what is typically taken. Other support from the institution could be targeted to those individuals. This approach is consistent with the view of Adams and Sasse (1999) who found that lack of cybersecurity knowledge and perceptions of risky behaviors as low risk could be viewed as the result of inadequate communication from institutional representatives to the users that they oversee. Institutions with high numbers of users with such victim profiles are encouraged to examine their communications to determine if improvements in communication can result in a reduction in numbers of users who fit victim profiles. Future research is needed to determine whether efforts to target individuals at high risk of being a cybersecurity victim with training or other support is effective in reducing their risk.

The present study also yielded some differences between men and women. As in prior research, men reported higher levels of sensation-seeking than women (Kennison et al., 2016) and higher levels of general risk-taking in daily life (Kennison et al., 2016). Nevertheless, we did not find that the level of self-reported risky cybersecurity behavior differed significantly. One prior study found that women engaged in risky cybersecurity behavior significantly less often than men (Anwar et al., 2017). Men reported significantly higher levels of password security knowledge. Prior research in which participants were drawn from employment settings have not observed differences in security knowledge, attitude, and behavior (McCormac et al., 2017). Prior research carried out on the online platform Amazon’s Mechanical Turk (MTurk) also found that men reported significantly higher levels of knowledge than women (Cain et al., 2018).

There are multiple weaknesses of the research, including the characteristics of our sample. Our sample was majority female (i.e., 63.9%), relatively young (19.46 years on average), and drawn from university students enrolled in psychology and speech communication courses, which typically enroll more women than men. Our participants may be less aware of cybersecurity issues than others who are older or who are drawn from other settings. For this reason, the results may not generalize to other populations. A second limitation is that our assessments of risky cybersecurity behaviors and secure password knowledge were created for this study, and although the items for each construct demonstrated high internal consistency, they may fail to capture all aspects of risky cybersecurity behavior and/or secure password knowledge. The questions that we used to assess knowledge may have tapped into overlapping topics and may have reflected participants’ opinion about their knowledge rather than actual knowledge. A third limitation is that we measured self-reported knowledge and cybersecurity behavior from participants. We may have observed different results had we been able to assess participants’ knowledge and behavior using different methods. Future research is needed to determine whether our results are replicated in other samples and/or other populations. A fourth limitation may be the fact that the research was carried out in an online survey. It is possible that different results may be obtained when face-to-face survey methodologies are used.

Future research on this topic may improve on the present research in a number of ways. In the present study, we developed six-item questionnaire to assess behavior in situations familiar to college students and four-item questionnaire to assess participants’ opinion about their cybersecurity knowledge. Future research could improve on the present research by assessing cybersecurity knowledge and behavior using objective measures instead of or in addition to self-report measures. Participants’ responses to our questions about knowledge and behavior may not accurately measure either construct, but reflect a mixture of each construct and opinion, which may have led to participants responding in ways that they perceived to be socially more desirable. Future research could include measures to assess social desirability responding (e.g., Crowne and Marlowe, 1960). Future research could also include a wider variety of question types, such as open-ended questions that enable the researcher to assess participants’ prior experiences (i.e., good or bad) with cybersecurity as well as other topics. The present research did not include open-ended questions about participants’ past cybersecurity experiences.

In summary, the research showed that taking into consideration sensation-seeking personality traits and general risk-taking in daily life, in addition to participant sex, Big Five personality traits, and knowledge about security passwords accounts for about 34% of variance in risky cyber security behaviors. From previous work, this is one of the highest amounts of variance accounted for in cyber security behaviors. This greatly reinforces that more research is needed on the relationship between personality traits (or other traits) and cyber security behaviors. Institutions who rely on training to increase awareness about cybersecurity issues as a means to reduce risky cybersecurity behaviors may find that using personal characteristics to target training to individuals who are the most likely to engage in risky behaviors may lead to better return on investment. Some individuals are more likely to engage in risky cybersecurity behaviors than others. Better personalized cybersecurity training is needed from organizations to improve the cybersecurity compliance and cybersecurity behaviors of individuals.

## Data Availability Statement

The raw data supporting the conclusions of this article will be made available by the authors, without undue reservation.

## Ethics Statement

The studies involving human participants were reviewed and approved by Oklahoma State University Institutional Review Board (IRB). Written informed consent for participation was not required for this study in accordance with the national legislation and the institutional requirements.

## Author Contributions

SK and EC-T formulated the idea for the study, constructed items for the survey, and contributed to the writing of the manuscript. SK conducted the statistical analyses. Both authors contributed to the article and approved the submitted version.

## Conflict of Interest

The authors declare that the research was conducted in the absence of any commercial or financial relationships that could be construed as a potential conflict of interest.
